# Normal values for ^18^F-FDG uptake in organs and tissues measured by dynamic whole body multiparametric FDG PET in 126 patients

**DOI:** 10.1186/s13550-022-00884-0

**Published:** 2022-03-07

**Authors:** André H. Dias, Allan K. Hansen, Ole L. Munk, Lars C. Gormsen

**Affiliations:** 1 Department of Nuclear Medicine and PET Centre, Aarhus University Hospital, Palle Juul-Jensens Boulevard 165, 8200 Aarhus, Denmark; 2 Department of Clinical Medicine, Aarhus University, Aarhus, Denmark

**Keywords:** Dynamic whole-body PET, Multiparametric imaging, Normal values, Patlak

## Abstract

**Background:**

Dynamic whole-body (D-WB) FDG PET/CT is a recently developed technique that allows direct reconstruction of multiparametric images of metabolic rate of FDG uptake (MR_FDG_) and “free” FDG (DV_FDG_). Multiparametric images have a markedly different appearance than the conventional SUV images obtained by static PET imaging, and normal values of MR_FDG_ and DV_FDG_ in frequently used reference tissues and organs are lacking. The aim of this study was therefore to: (1) provide an overview of normal MR_FDG_ and DV_FDG_ values and range of variation in organs and tissues; (2) analyse organ time-activity curves (TACs); (3) validate the accuracy of directly reconstructed MR_FDG_ tissue values versus manually calculated *K*_*i*_ (and MR_FDG_) values; and (4) explore correlations between demographics, blood glucose levels and MR_FDG_ values. D-WB data from 126 prospectively recruited patients (100 without diabetes and 26 with diabetes) were retrospectively analysed. Participants were scanned using a 70-min multiparametric PET acquisition protocol on a Siemens Biograph Vision 600 PET/CT scanner. 13 regions (bone, brain grey and white matter, colon, heart, kidney, liver, lung, skeletal muscle of the back and thigh, pancreas, spleen, and stomach) as well as representative pathological findings were manually delineated, and values of static PET (SUV), D-WB PET (*K*_*i*_, MR_FDG_ and DV_FDG_) and individual TACs were extracted. Multiparametric values were compared with manual TAC-based calculations of *K*_*i*_ and MR_FDG_, and correlations with blood glucose, age, weight, BMI, and injected tracer dose were explored.

**Results:**

Tissue and organ MR_FDG_ values showed little variation, comparable to corresponding SUV variation. All regional TACs were in line with previously published FDG kinetics, and the multiparametric metrics correlated well with manual TAC-based calculations (r^2^ = 0.97, *p* < 0.0001). No correlations were observed between glucose levels and MR_FDG_ in tissues known not to be substrate driven, while tissues with substrate driven glucose uptake had significantly correlated glucose levels and MR_FDG_ values.

**Conclusion:**

The multiparametric D-WB PET scan protocol provides normal MR_FDG_ values with little inter-subject variation and in agreement with manual TAC-based calculations and literature values. The technique therefore facilitates both accurate clinical reports and simpler acquisition of quantitative estimates of whole-body tissue glucose metabolism.

**Supplementary Information:**

The online version contains supplementary material available at 10.1186/s13550-022-00884-0.

## Introduction

Positron emission tomography (PET) using ^18^F-fluorodeoxyglucose (FDG) is a key part of diagnostics and follow-up of both malignant and non-malignant diseases. Conventional static FDG PET routine consists of a single whole-body (WB) pass and reconstruction of a standard uptake value (SUV) image representing the tracer distribution 60 min after tracer injection [1]. This snapshot of accumulated tracer is not without problems. SUV images are highly dependent on the timing of the scan with any delay incurring a variation of measured activity. Factors such as scanner calibration, non-perfect injections and even the subject’s body composition or blood glucose levels also influence the resulting images [2, 3]. Furthermore, static FDG PET images capture not only FDG-6-P retained in glucose consuming tissue but also a substantial background of unbound FDG in tissue and circulation, complicating the evaluation of vascularized organs.

Advances in PET scanner technology and software have introduced new possibilities [4, 5]. Dynamic whole-body (D-WB) PET/CT is a recently developed technique involving multiple WB passes and extraction of an arterial image-derived input function (IDIF), which provide the dynamic PET data needed for direct reconstruction of WB multiparametric images based on the linear Patlak analysis [6, 7]. Multiparametric imaging complements the standard SUV image with two new parametric images: One representing the metabolic rate of FDG into the tissue (MR_FDG_) and another representing the distribution volume of free FDG in the tissue (DV_FDG_).

Until now, there is sparse data regarding clinical applications of D-WB PET [8, 9]. A recent study published by our group demonstrated the feasibility of the technique in a clinical setting and also showed some promising results from the resulting MR_FDG_ and DV_FDG_ images including superior lesion target-to-background ratios as well as fewer false positive findings [8]. As multiparametric imaging becomes available in more PET facilities and in all PET/CT systems, nuclear medicine clinicians will need to familiarize themselves with MR_FDG_ and DV_FDG_ images. In particular, organ and tissue thresholds for physiological MR_FDG_ and DV_FDG_ values should be established to ease detection of pathology.

We therefore performed a review of our clinically acquired D-WB FDG PET scans to provide estimates of expected normal values and population-based variation of SUV, MR_FDG_ and DV_FDG_ in selected tissues and organs. In addition, we extracted organ and tissue time-activity curves (TACs), validated the multiparametric MR_FDG_ and DV_FDG_ values obtained by direct reconstruction against post-reconstruction manual TAC-based calculations and explored correlations between age, diabetes status, body composition and the multiparametric values.

## Materials and methods

### Patient population

This was a retrospective analysis of prospectively collected data. Included subjects were recruited from the entire cohort of patients referred for FDG PET/CT as part of their clinical diagnostic work-up or treatment response evaluation. Inclusion into the main D-WB protocol and study was solely based on whether patients were deemed fit to lie still for 70 min while in the PET/CT scanner. The study was approved by the local ethics committee in Region Midtjylland (1-10-72-188-19).

D-WB data from 126 individuals (100 patients without diabetes (Non-DM) and 26 patients with diabetes (DM)) were analysed. The study population’s indications for PET referral, sex and age are shown in Table 1.

**Table 1:** PET study indication and population demographics

Scan indication		Number of patients			Age distribution*		
		Total	Male	Female	Total	Male	Female
Cancer of unknown primary origin	Non-DM	6	1	5	54.5 [35–65]	53	54.8 [35–65]
	DM	2	1	1	67 [59–75]	75	59
Gastro-intestinal cancer	Non-DM	3	2	1	57.3 [45–68]	63.5 [59–68]	45
	DM	1	0	1	57	–	57
Head and neck cancer	Non-DM	15	7	8	58.6 [23–81]	54.7 [23–81]	62 [49–72]
	DM	6	2	4	66.6 [55–77]	73.5 [70–77]	63.3 [55–70]
Infection and inflammation	Non-DM	22	13	9	50.4 [22–76]	49.1 [22–71]	52.2 [24–76]
	DM	5	3	2	60.6 [51–74]	59.3 [52–64]	62.5 [51–74]
Lung cancer	Non-DM	38	18	20	64.8 [49–81]	66 [49–78]	63.7 [51–81]
	DM	7	6	1	73.4 [69–77]	74.2 [72–77]	69
Lymphoma	Non-DM	16	9	7	55.5 [18–85]	53.6 [18–85]	58 [37–72]
	DM	3	3	0	51.7 [28–64]	51.7 [28–64]	–
Uro-genital cancer	Non-DM	0	0	0	–	–	–
	DM	2	1	1	69 [63–75]	75	63
Total	Non-DM	100	50	50	58.4 [18–85]	57.4 [18–85]	59.3 [24–81]
	DM	26	16	10	65.4 [28–77]	67.2 [28–77]	62.6 [51–74]

*Values presented as mean [range]

### Data acquisition and image reconstruction

Participants were scanned using a fully automated multiparametric PET/CT acquisition protocol (FlowMotion® Multiparametric PET, Siemens Healthineers, Knoxville, USA) on a Siemens Biograph Vision 600 PET/CT scanner (Siemens Healthineers, Knoxville, USA) with 26.2 cm axial field of view. In short, a 70-min multiparametric PET acquisition protocol was started at the time of a standardized injection of FDG (4 MBq/kg) using an Intego PET Infusion System (MEDRAD, Inc., Warrendale, PA, USA). The PET protocol consisted of 1) a 6-min dynamic scan with the bed fixed at the chest region including organs as heart, liver and most importantly aorta, and 2) a 64-min dynamic WB PET scan consisting of 16 continuous bed motion passes: 7 × 2-min WB passes followed by 9 × 5-min WB passes.

The automated multiparametric scan protocol automatically identified the aorta on the low-dose WB CT scan [10] and placed a VOI (1.6 mm^3^ cylinder) to extract the IDIF from the full dynamic PET series of the chest region. Such IDIF is robust and can be used to replace an arterial input function (AIF) for quantitative Patlak modelling [11].

**Fig. 1:**
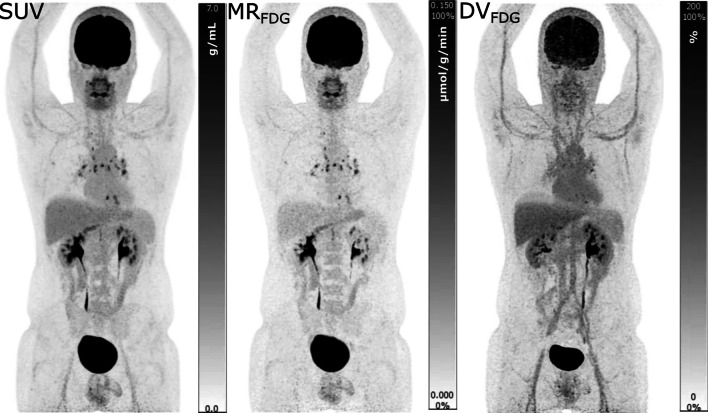
Example of SUV and Patlak images analysed in our study. In this case, a 34-year-old man with mediastinal sarcoidosis. Left column: static SUV images; Middle column: MR_FDG_ images; right column: DV_FDG_ images. The actual FDG uptake in the inflammatory sarcoid lymph nodes is evident on the MR_FDG_ image, whereas the free FDG in the circulation is clearly visible on the DV_FDG_ image

Multiparametric images (MR_FDG_ and DV_FDG_) were reconstructed using data from 40 to 70 min post injection (p.i.) and the IDIF. A standard-of-care static SUV image was reconstructed using data from 60 to 70 min p.i. (see example in Fig. 1). A more detailed description of the D-WB PET acquisition technique and image reconstruction parameters can be found in our previous publication [8].

### Interpretation of multiparametric WB FDG images

The Patlak model is currently the only model available for direct reconstruction of WB parametric images, but the use of the same irreversible model in the entire image does lead to a challenging image interpretation.

Assuming irreversible kinetics, *K*_*i*_ represent the steady-state rate of tracer uptake in the trapped compartment representing FDG-6-phosphate. For ^18^FDG, *K*_*i*_ can be multiplied by the subject’s blood glucose to obtain the metabolic rate of ^18^FDG, MR_FDG_ = *K*_*i*_ × blood glucose, which can be divided with the lumped constant to obtain the metabolic rate of glucose, MR_Glu_ = MR_FDG_/LC. The images and tables have not been corrected for LC, i.e. we used LC = 1, as it varies by tissue type (see Additional file 1: table S3 for a list of published values) and is not known for all tissues. Therefore, as of today, it is not really possible to generate a meaningful full WB parametric image of MR_Glu_, and for that reason we do not present these values. However, for tissues with well-established LC values, such as brain [12, 13], MR_FDG_ can easily recalculated into MR_Glu_, e.g. for comparison with single-organ studies where LC have been used.

The apparent distribution volume, DV_FDG_, is approximately equal to the sum of the blood volume and distribution volume of the reversible components, which both varies between tissues and cannot be separated by the Patlak model [14]. A separate estimate of the blood volume would require full kinetic modelling. Thus, DV_FDG_ reflects the combined distribution volume of free ^18^FDG in blood and tissue.

Clear interpretations of MR_FDG_ and DV_FDG_ values are based on the assumption of *irreversible* ^18^FDG kinetics. However, this assumption is not always met with notable exceptions as the liver and kidney. Thus, in multi-parametric WB images of MR_FDG_ and DV_FDG_ there are tissues with *reversible* FDG kinetics that obscure the physiologic interpretation of the voxel values. As an example, the liver is known to de-phosphorylate ^18^FDG-6P, and this reversible FDG kinetics led to lower MR_FDG_ values (less irreversible trapping) and higher DV_FDG_ (the reversible compartments include FDG-6P).

### Image analysis and VOI delineation

We analysed 13 volumes of interests (VOIs) corresponding to different organ and tissue locations: bone, brain (white and grey matter), colon, heart, kidney, liver, lung, muscle (paravertebral and thigh), pancreas, spleen and stomach. Due to the differing morphologies of the selected organs and tissues, different approaches were used to outline representative VOIs, and care was taken to ensure that the outlined VOIs corresponded to areas not affected by pathology or visual artefacts. See Additional file 1: table S1 for the methodology used in each region.

Furthermore, we also analysed an area representative of pathology in these patients. In oncological patients, the VOI was placed on the primary tumour, while for patients referred for infectious or inflammatory disease, a VOI was placed in the area of greatest FDG uptake. See Additional file 1: table S1 for the methodology used. Excluded from this analysis were patients who had previously undergone surgery or who had been treated with chemo-/radiotherapy. Also excluded were patients where no signs of active disease were detected on the PET scan. The final analysis of pathological findings was therefore performed on 55 patients.

Multiparametric images were visually inspected using Hermes Gold Client v.2.5.0 (Hermes Medical Solutions AB, Stockholm, Sweden). VOI delineation of the multiparametric images was performed by AHD using PMOD® 4.0 (PMOD Technologies Ltd, Zürich, Switzerland). Semiquantitative values of SUV_max_ and SUV_mean_ were obtained from the conventional PET reconstructions, whereas MR_FDG_ and DV_FDG_ values were extracted from the multiparametric images.

### Time-activity curves

TACs were obtained from the 6-min dynamic scan of the chest region and the 16 D-WB passes. Analyses of brain regions were limited to patients scanned from the top of the skull. Therefore, the analysis of the grey and white matter was performed in a total of 75 patients (14 DM and 61 non-DM). The VOIs used for the manual TAC-based calculation in PMOD were the same that had been delineated and used to extract parameter values from multiparametric images.

### Comparison of multiparametric and manual TAC-based MRFDG and DVFDG values

Kinetic parameter estimates derived from post-reconstruction TAC-based analyses can be biased compared to those from direct reconstruction of parametric images [15]. Thus, we compared kinetic parameter estimates using the two methods. From a subpopulation of 10 randomly selected patients, the D-WB series and IDIF were used to manually calculate MR_FDG_ and DV_FDG_ values. These TAC-based parameters were estimated by linear Patlak analysis in PMOD® 4.0, using the PKIN module. Measured blood sugar levels were used to calculate MR_FDG_. TAC-based MR_FDG_ and DV_FDG_ values were subsequently compared with values extracted from multiparametric images obtained using direct reconstruction using the FlowMotion® Multiparametric PET software from Siemens Healthineers.

### Statistical analysis

The statistical analysis was performed using Stata 16 or GraphPad Prism 9.2.0. Statistical tests were used for group comparisons based on whether data were normally distributed and paired/unpaired. Pearson’s correlation analysis was performed for the relation between MR_FDG_ and SUV values and blood glucose, patient age, weight, tracer dose and BMI. *p* values of < 0.05 were considered significant. Continuous group data are presented as mean ± SD or median (range) as appropriate. Time-series are presented as mean ± SEM.

## Results

### Organ and tissue quantitative and semi-quantitative FDG uptake

Normal values of SUV_mean_, SUV_max_, MR_FDG mean_, *K*_*i*,mean_, and DV_FDG,mean_ (Table 2) were obtained for the bone, brain grey and white matter, colon, heart, kidney, liver, lung, skeletal muscle, pancreas, spleen and stomach. The same values were also obtained for the subpopulations of male and female patients (patients without diabetes Additional file 1: table S2A, patients with diabetes Additional file 1: table S2B).

**Table 2:** Normal values of the population without diabetes (*N* = 100) and the population with diabetes (*N* = 26)

Volume of interest		SUV_mean_* (g/mL)	SUV_max_* (g/mL)	100 × MR_FDG_* (µmol/g/min)	100 × *K*_*i*_ * (mL/mL/min)	DV_FDG_* (%)
Bone	Non-DM	1.90 [1.12–4.87]	2.72 [1.59–6.12]	3.69 [1.08–9.09]	0.67 [0.16–1.57]	30.97 [3.50–51]
	DM	1.85 [1.12–2.61]	2.79 [1.66–4.24]	4.28 [0.64–90]	0.63 [0.09–1.03]	23.51 [6.24–60.12]
Brain GM^†^	Non-DM	8.08 [4.85–13.56]	15.20 [8.66–46.50]	17.46 [11.68–27.61]	3.28 [2.02–5.40]	81.18 [13.58–140.70]
	DM	6.69 [4.65–9.43]	11.60 [8.59–19.28]	16.80 [12.79–24.28]	2.23 [1.62–3.92]	68.79 [41.6–95.26]
Brain WM^†^	Non-DM	3.39 [2.09–5.29]	5.86 [3.59–10.30]	6.03 [4.02–9.53]	1.09 [0.72–1.80]	46.07 [15.29–87.4]
	DM	2.94 [2.17–3.68]	4.88 [3.63–6.68]	6.44 [4.20–8.27]	0.86 [0.55–1.33]	39.76 [29.12–67.61]
Colon	Non-DM	1.26 [0.52–6.36]	2.38 [0.95–12.2]	3.17 [1.24–21.23]	0.57 [0.23–3.60]	47.70 [17.45–174.4]
	DM	1.84 [0.87–5.72]	3.17 [1.49–10.48]	6.07 [0.68–20.55]	0.74 [0.10–2.19]	56.46 [35.80–163.7]
Heart	Non-DM	4.48 [0.72–14.50]	9.53 [1.58–33.6]	11.03 [0.35–42.6]	1.82 [0.08–7.22]	54.17 [16.84–191.70]
	DM	2.67 [0.99–13.24]	7.01 [2.06–30.97]	5.90 [0.14–43.46]	0.86 [0.02–6.30]	48.50 [28.53–123.80]
Kidney	Non-DM	2.03 [0.67–3.03]	3.55 [1.32–4.77]	3.81 [0.08–7.95]	0.69 [0.01–1.45]	95.54 [32.51–141]
	DM	2.31 [1.91–3.18]	4.02 [3.11–6.05]	4.49 [0.53–8.38]	0.67 [0.08–1.10]	99.98 [75–137.70]
Liver	Non-DM	2.26 [1.31–3.11]	3.80 [2.51–5.69]	2.02 [0.74–4.35]	0.36 [0.12–0.73]	83.80 [43.66–126]
	DM	2.19 [1.77–2.81]	3.84 [2.89–5.17]	2.76 [0.15–5.35]	0.35 [0.03–0.54]	80.43 [62.71–105.90]
Lung	Non-DM	0.40 [0.07–0.83]	0.83 [0.34–1.46]	0.35 [0.03–1.74]	0.06 [0.01–0.30]	15.18 [2.13–28.15]
	DM	0.41 [0.15–0.62]	0.84 [0.5–1.19]	0.51 [0.05–0.97]	0.07 [0.01–0.11]	15.01 [6.11–21.34]
Muscle back	Non-DM	0.65 [0.38–1.01]	1.10 [0.76–1.99]	0.89 [0.40–1.64]	0.16 [0.08–0.30]	15.19 [6.02–33.72]
	DM	0.69 [0.41–1.17]	1.23 [0.73–2.16]	1.16 [0.08–2.68]	0.14 [0.01–0.38]	15.89 [7.67–29.84]
Muscle thigh	Non-DM	0.55 [0.36–1.41]	1.19 [0.72–3.11]	0.98 [0.14–2.28]	0.17 [0.03–0.39]	13.41 [6.26–25.80]
	DM	0.54 [0.38–2.02]	1.26 [0.81–4.39]	1.06 [0.10–5.11]	0.14 [0.02–0.82]	15.33 [11.16–30.23]
Pancreas	Non-DM	1.68 [1.15–2.44]	2.71 [1.64–4.29]	3.40 [1.16–6.67]	0.62 [0.26–1.15]	68.72 [42.57–123.80]
	DM	1.89 [1.29–2.43]	2.83 [2.04–3.43]	4.62 [0.81–6.09]	0.62 [0.12–0.80]	70.05 [42.48–92.01]
Spleen	Non-DM	1.96 [1.41–5.93]	2.83 [1.98–8.46]	2.45 [1.18–15.30]	0.44 [0.20–2.78]	58.30 [10.07–111.60]
	DM	1.91 [1.55–2.20]	2.90 [2.19–3.69]	2.55 [0.37–5.41]	0.35 [0.05–0.62]	54.30 [41.57–87.58]
Stomach	Non-DM	2.26 [1.33–5.25]	4.19 [2.43–9.72]	5.88 [2.84–19.13]	0.99 [0.49–3.24]	79.63 [45.22–178.40]
	DM	2.20 [1.47–2.82]	4.07 [2.79–5.38]	6.87 [0.94–11.14]	0.94 [0.14–1.41]	78.38 [40.73–102.80]

*Values are median [min–max]; † Brain VOIs: Non-DM N = 61; DM *N* = 14. Note that MR_FDG_ and *K*_*i*_ values are multiplied by 100

Lumped constant = 1. See Additional file 1: table S3 for information on the use of a tissue adjusted lumped constant for the calculation of MR_FDG_

SUV_max_ is included for comparison, as it is the most used PET metric in clinical practice

**Fig. 2:**
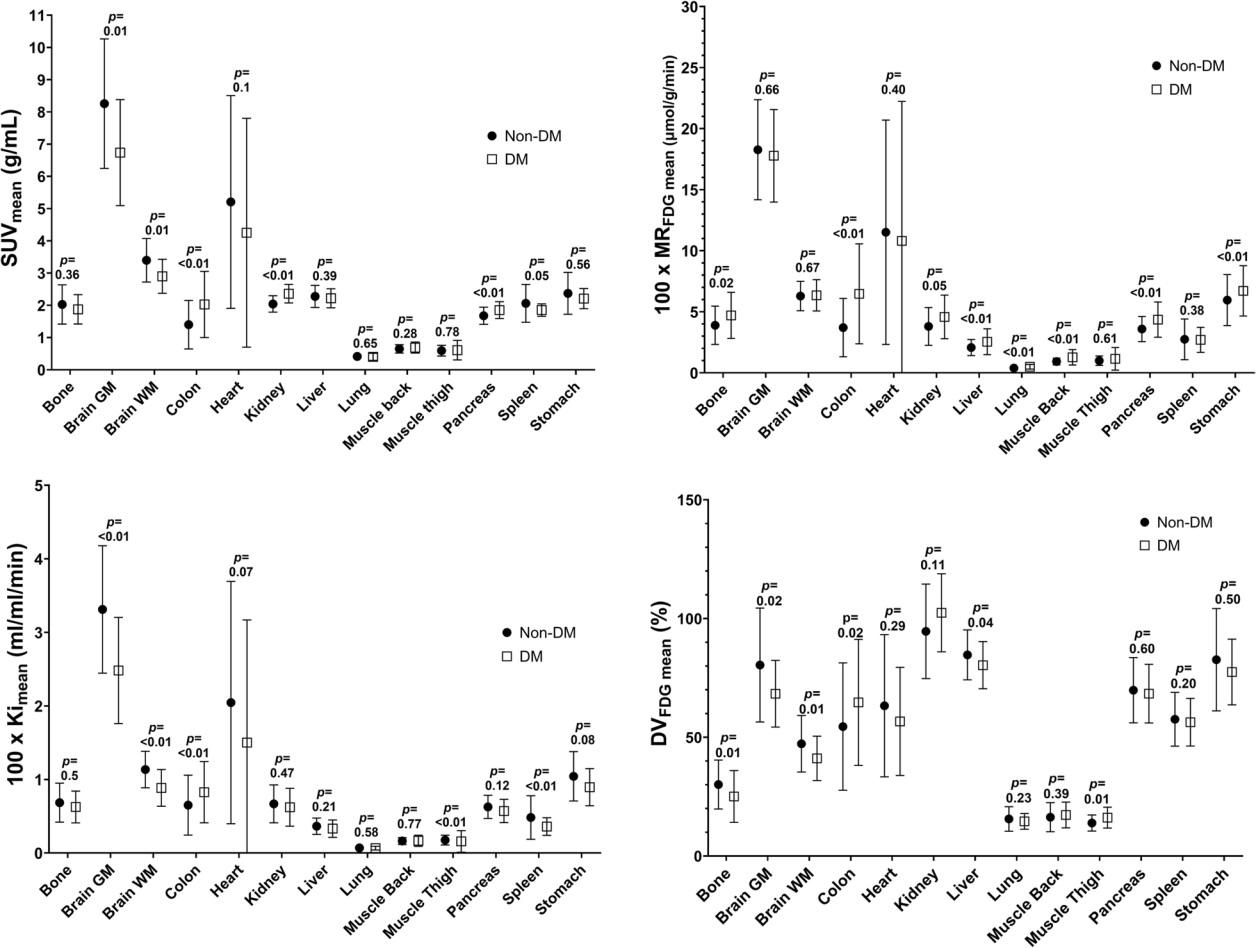
Distribution plots of SUV_mean_, MR_FDG,mean_, *K*_*i*,mean_ and DV_FDG,mean_ for the DM and Non-DM populations in 13 different types of tissue and organs. Plotted are the mean and standard deviation values. As seen, population variation was remarkably similar for SUV and MR_FDG_ values. Calculated *p*-values between patients with DM and without DM are displayed above each organ

SUV_mean_ and MR_FDG,mean_ distribution plots are presented in Fig. 2. There was a remarkable similarity between the plots underlining the robustness of the quantitative values derived from the multiparametric images. Unsurprisingly, the organs that displayed the greatest MR_FDG_ and by inference glucose metabolism were the heart and the grey matter of the brain, whereas the lowest measured glucose metabolism was observed in the lungs, an area of low tissue fraction.

SUV, MR_FDG_ and DV_FDG_ values for different pathologies are displayed in Table 3. MR_FDG_ values were significantly greater in most pathological lesions than in background tissue. However, no difference was observed between malignant and non-malignant pathological lesions.

**Table 3:** Values from pathological findings (*N* = 55)

Volume of Interest	*N*	SUV_mean_* (g/mL)	SUV_max_* (g/mL)	100 × MR_FDG_* (µmol/g/min)	100 × *K*_*i*_ * (mL/mL/min)	DV_FDG_* (%)
Gastrointestinal cancer	4	8.83 [6.58–14.70]	15.52 [12.06–25.40]	23.53 [14.72–31.49]	4.31 [2.78–7.32]	244.70 [125–360.40]
Head and neck cancer	8	9.57 [5.19–22.00]	17.02 [9.69–39.57]	26.86 [11.47–68.31]	4.14 [2.12–11.02]	236.90 [88.61–620.30]
Infection and Inflammation	13	4.60 [2.54–9.33]	9.14 [4.67–17.88]	14.85 [3.59–38.44]	2.46 [0.84–6.41]	126.70 [50.98–230]
Lung adenocarcinoma	14	9.88 [1.38–24.3]	15.07 [2.47–35.52]	28.53 [4.05–77.55]	5.06 [0.53–14.36]	278.00 [43.38–762]
Lung squamous cell carcinoma	9	14.30 [7.33–28.60]	21.59 [2.45–44.48]	31.27 [16.84–55.55]	5.49 [3.01–10.48]	272.90 [114–592]
Lymphoma	7	7.40 [4.64–17.80]	13.49 [8.05–34.50]	23.13 [9.77–68.51]	4.03 [1.74–12.46]	248.80 [102.10–938]

*Values are median [min–max]

Note that MR_FDG_ and *K*_*i*_ values are multiplied by 100. Lumped constant = 1

SUV_max_ is included for comparison, as it is the most used PET metric in clinical practice

We next investigated the impact of diabetes on SUV, MR_FDG_ and DV_FDG_ values. As expected, patients with diabetes had significantly higher average blood glucose than patients with no history of diabetes (7.6 ± 1.5 mmol/L vs. 5.7 ± 0.7 mmol/L, *p* < 0.0001), whereas the two groups did not differ on age, sex and BMI distributions.

SUV_mean_ was significantly lower in patients with diabetes in both brain areas and the heart and was greater in the colon, kidney, pancreas and spleen. By contrast, MR_FDG_ in the brain and heart was not impacted by diabetes status, whereas patients with diabetes had significantly greater MR_FDG_ in the bone, colon, kidney, liver, lungs, paravertebral muscle, pancreas, and stomach.

### Time-activity curves

**Fig. 3:**
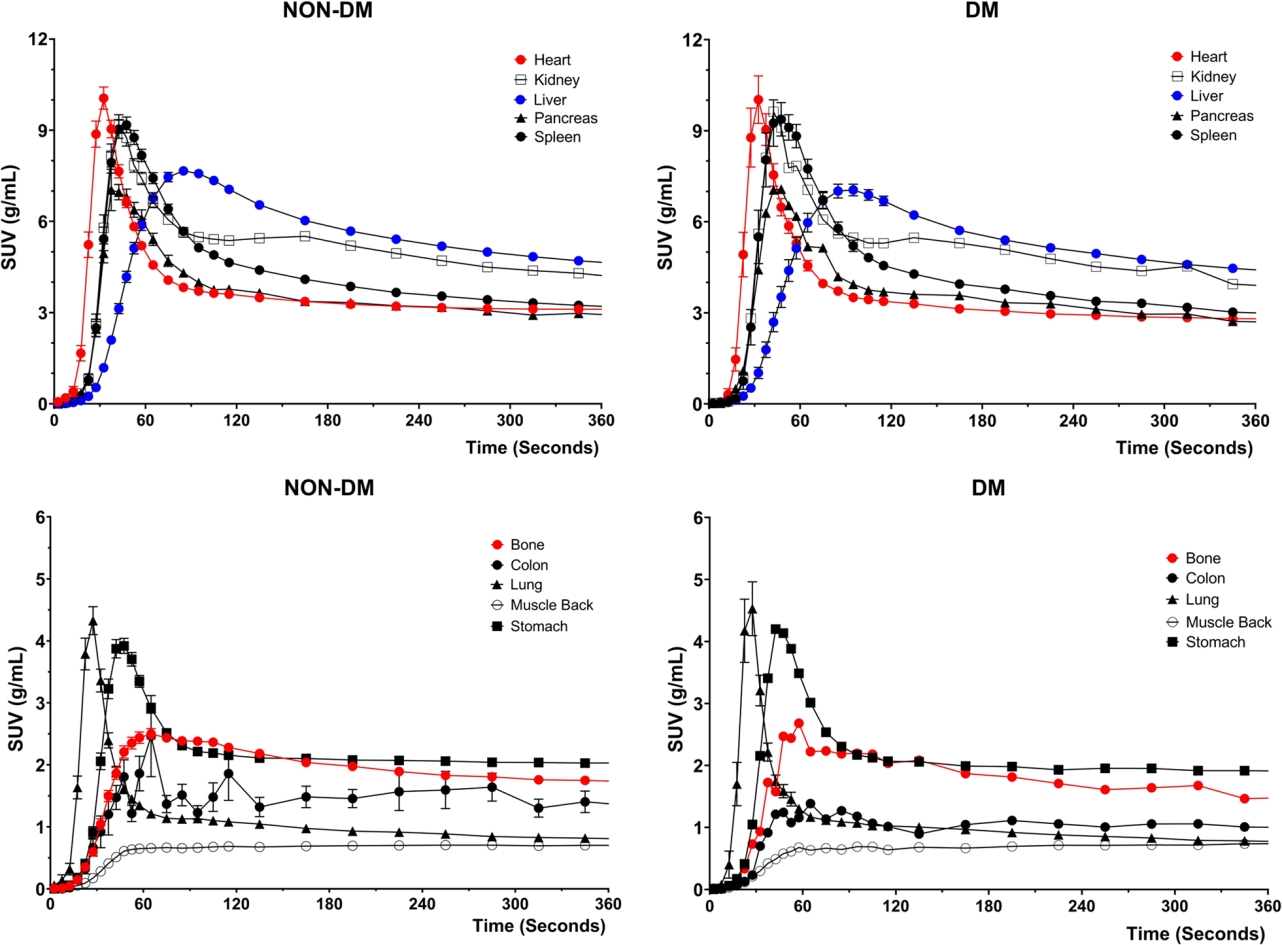
Time-activity curves for the first 6 min of dynamic scanning over the chest area. Represented values are mean and SEM of all 126 patients. The panels on the left side correspond to the patients without diabetes; the panels on the right side to the patients with diabetes

**Fig. 4:**
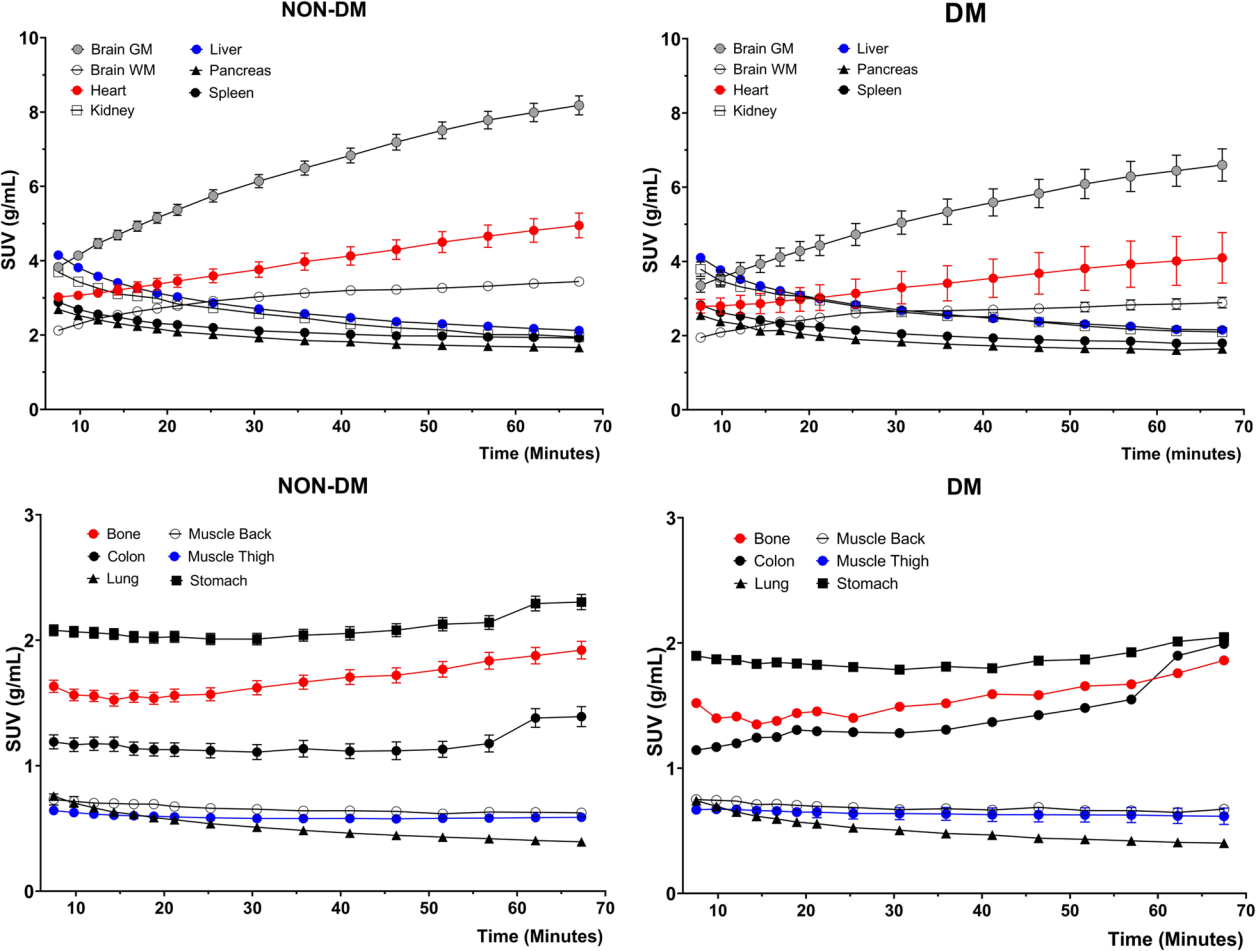
Time-activity curves for the remaining 6–70 min of dynamic WB scanning. Represented values are mean and SEM of all 126 patients. The panels on the left side correspond to the patients without diabetes; the panels on the right side to the patients with diabetes

The D-WB dynamic series contain complete TACs of most presented organs and tissues as presented in Figs. 3 and 4. The TACs confirm previous findings of FDG kinetics, such as the largely increasing uptake by the brain and heart, and decreasing uptake by the kidneys, but also interestingly solidly increasing FDG activity over time in the bone marrow. The characteristics of the TAC shapes are reflected in the SUV, MR_FDG_, and DV_FDG_ values in Fig. 2. Tissues with increasing TACs generally have greater MR_FDG_ values than tissue with decreasing TACs.

### Comparison of multiparametric and TAC-based MRFDG values

**Fig. 5:**
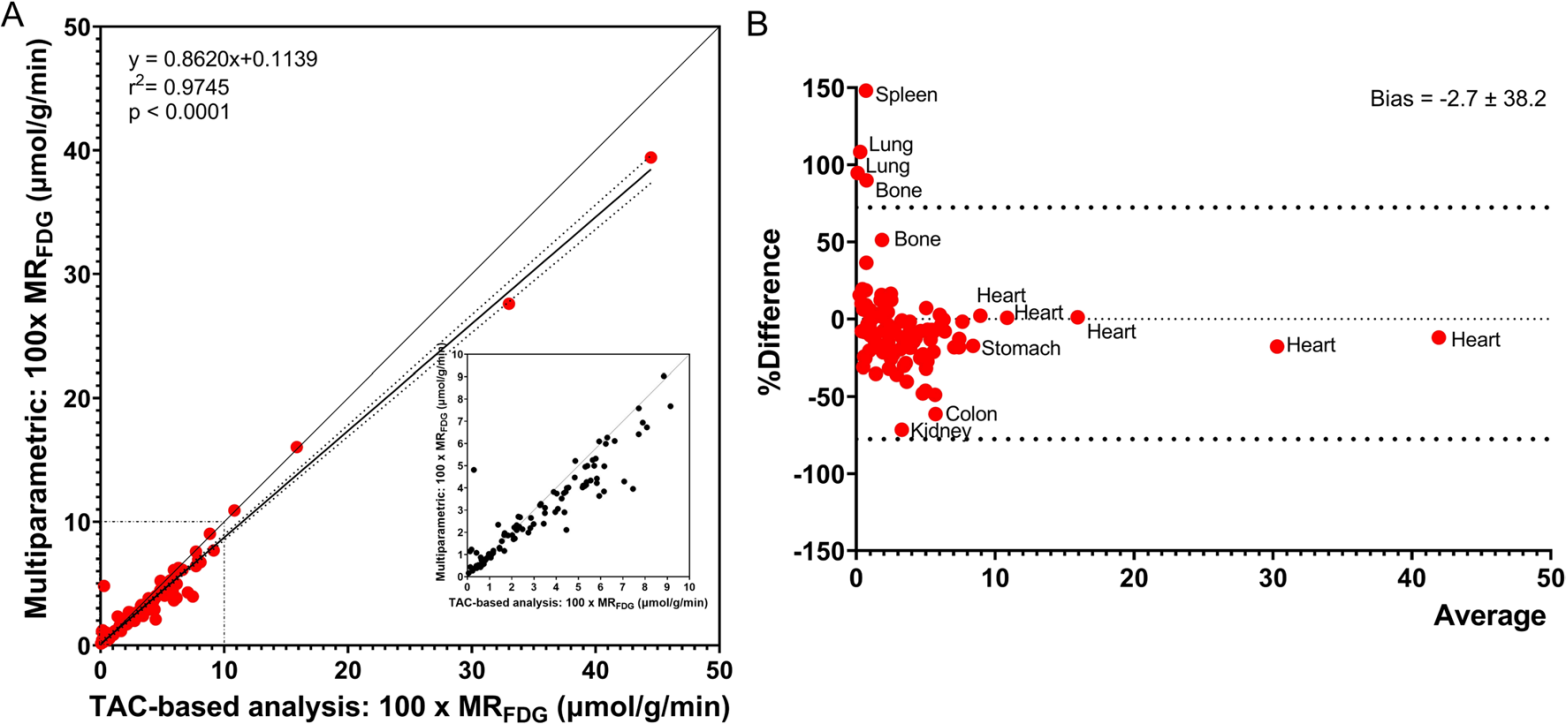
**A** Correlation between multiparametric MR_FDG_ values and manual TAC-based MR_FDG_ estimates using PMOD’s PKIN module. *N* = 10, corresponding to 110 correlation points. **B** Bland–Altman analysis of %Difference (100*(Multiparametric MR_FDG_ − TAC-based MR_FDG_)/average) vs average

Figure 5 shows the results of the comparison study between the values extracted from the multiparametric images from direct reconstruction versus the manual post-reconstruction TAC-based calculations of the Patlak model performed in PMOD’s PKIN module. The analysis showed an excellent correlation (*r*^2^ = 0.97, *p* < 0.0001), with a tendency towards higher manual TAC-based MR_FDG_ values (bias 3% in the Bland–Altman analysis).

### Correlations

**Fig. 6:**
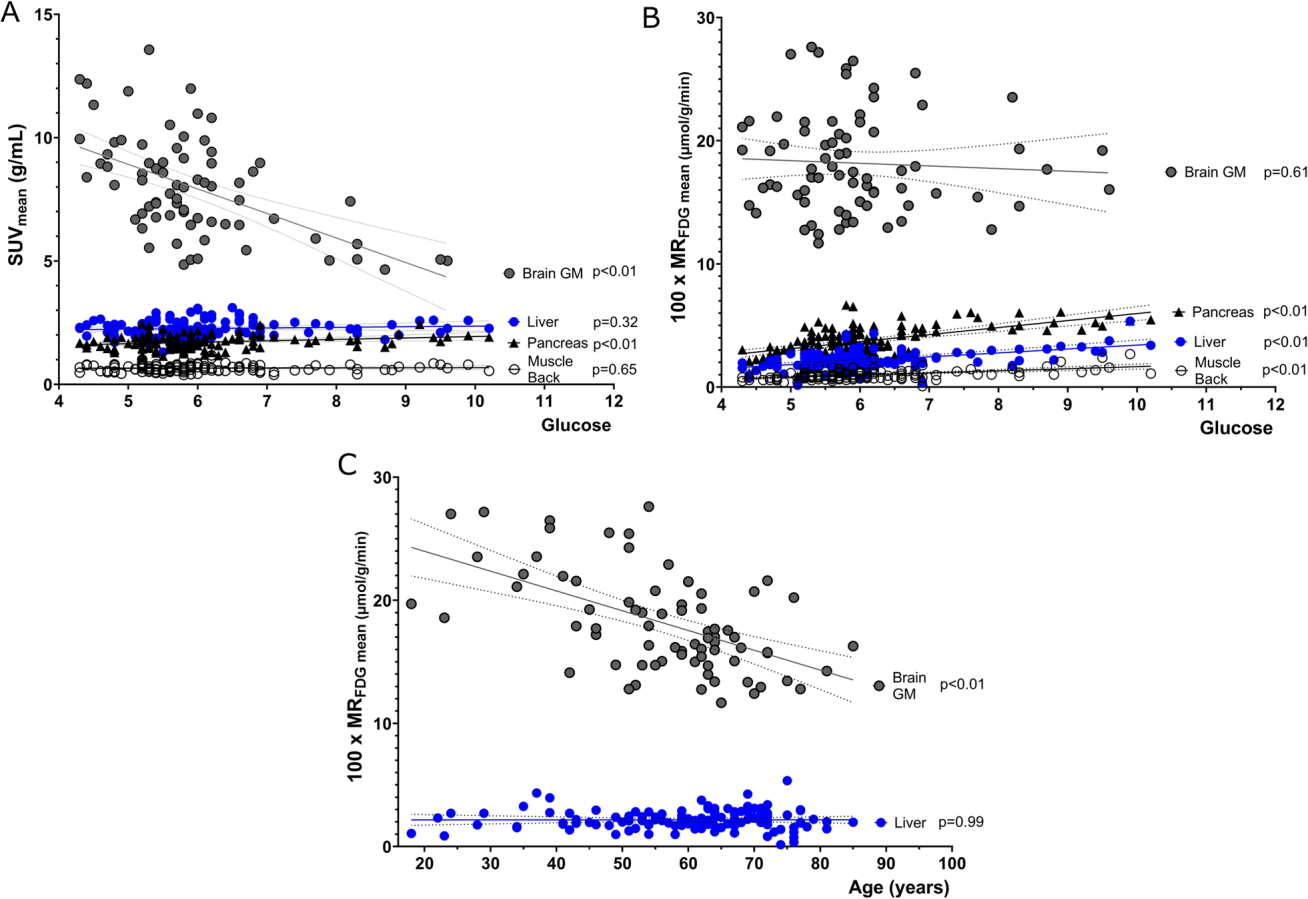
Correlation of **A** SUV_mean_ and **B** MR_FDG mean_ with glucose for brain grey matter, pancreas, liver, and skeletal paravertebral muscle. **C** Correlation of MR_FDG mean_ and age. Plotted are the VOIs for brain grey matter and liver tissue

**Fig. 7:**
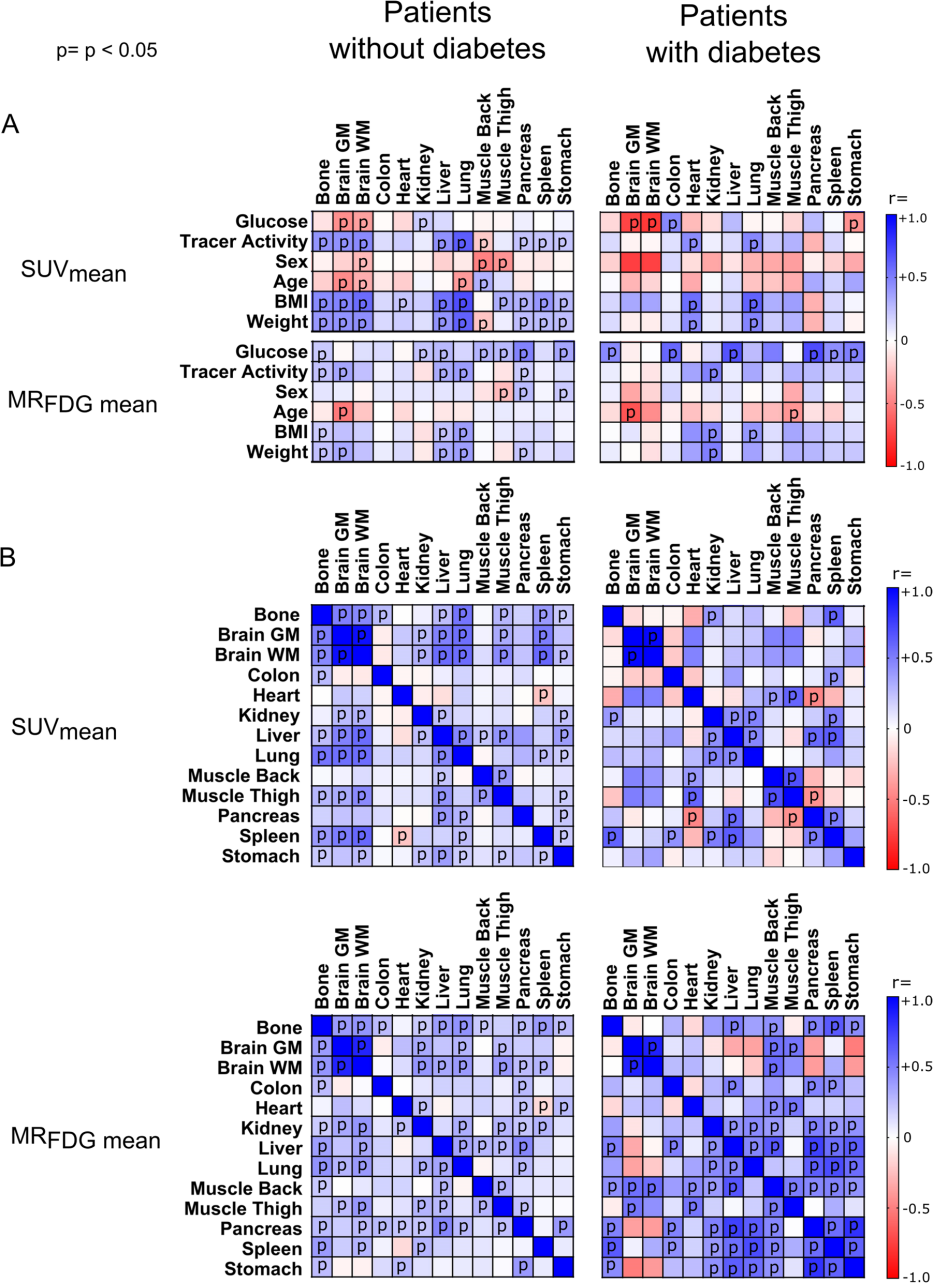
Pearson’s correlation analysis of patients with and without diabetes, of SUV_mean_ and MR_FDG mean_ with glucose levels, tracer activity, sex, age, BMI, and weight (**A**); as well as between paired sets of organs (**B**). Cells labelled “p” showed statistically significant correlation (*p* < 0.05) without correction for multiple comparisons. The full correlation results can be found in the Additional file 1: figure S1 and S2, table S4A, S4B, S5A and S5B

The brain and heart were the only organs where MR_FDG_ was not directly correlated with blood glucose (Fig. 6B). By contrast, brain SUV values were negatively correlated with blood glucose (Fig. 6A). Both cardiac and cerebral MR_FDG_ significantly decreased with increasing age as previously reported in numerous studies (Fig. 6C). Explorative correlations of SUV and MR_FDG_ with patient glucose levels, age, injected tracer dose and patient weight/BMI are presented in Fig. 7.

## Discussion

In daily clinical practice, static PET image evaluation consists fundamentally of a visual analysis that relies on good image quality and adequate contrast between target and background areas. Semiquantitative measurements of SUV, sometimes reported as a SUV ratio normalized to the blood or liver background, are often used to complement the visual information and are of particular relevance in clinical follow-up and response evaluation.

However, the use of SUV as a reference measurement has a several methodological limitations which can inhibit the correct interpretation of FDG PET/CT images. This is particularly relevant in treatment response evaluation studies as any delay in the scan acquisition time will result in different measurements [2]. Furthermore, these measurements are also dependent on factors such as hardware calibration, imaging protocols and patient physical characteristics [3, 16–18], meaning that it has so far proved impossible to reach international agreement on the use of FDG uptake levels to define pathology. Nevertheless, decades of optimization have been applied to conventional static SUV images to achieve the current standard observed in departments worldwide.

Contrasting this, multiparametric images from D-WB PET are novel in clinical PET and have yet to be optimized and standardized to the same extent [4]. Compared to SUV images, parametric images have different organ signal and lesion-to-background patterns [8, 9]. Thus, nuclear medicine clinicians therefore must update their reporting practices and measurements. The main purpose of this study was therefore to facilitate easier parametric image reading by reporting extensive parametric normal values of non-diseased tissue and organs.

It should be noted that the multiparametric images analysed in this study were produced by direct reconstruction from PET raw data [19], which lead to parametric images that are clearly less noisy than parametric images produced by the traditional indirect image-based approach [4]. Thus, the multiparametric images were of good visual quality and the distribution plots of mean organ and tissue MR_FDG_ were almost similar to that of conventional SUV values reflecting that there was little inter-individual variation in the overall presentation of organs and tissues in the parametric images. A narrow range of mean MR_FDG_ in background tissue and organs naturally enhances the detection of patterns of pathology and thus the clinical value of the parametric images. MR_FDG_ in pathological lesions was also uniformly greater than in background tissue as seen in Table 3.

However, a few caveats should be mentioned. First, although great care was observed during delineation of organ and tissue structures for the quantitative analysis, some of the resulting VOIs were drawn based on poor target-to-background radioactivity and difficult CT morphology. This was particularly evident for the pancreas and other abdominal organs. Second, the length of the acquisition protocol makes D-WB PET/CT images prone to motion artefacts, which profoundly impacts quantitative measurements such as MR_FDG_. Bulk motion tends to result in blurry images with underestimated SUV values in static PET imaging, whereas it may dramatically affect MR_FDG_ and DV_FDG_ values in areas adjoining FDG avid structures. The effect can cause artefacts at the edges of organs and around lesions. Third, overlapping signal from neighbouring structures proved to be impossible to avoid in all tissues. For example, the area of the pancreas is almost impossible to correctly individualize due to poor background radioactivity and high tissue density in the upper abdomen. It is expected that the values obtained from this region includes a degree of *spill-in* from neighbouring structures.

MR_FDG_ and DV_FDG_ values of the multiparametric images could be accurately reproduced by a traditional TAC-based Patlak analysis using PMOD PKIN, and a strong correlation between parameter estimates from the two methods was observed. However, a 3% bias towards greater MR_FDG_ estimates was present when calculated with the TAC-based Patlak analysis, which could be a consequence of the different noise propagation in the two methods: the multiparametric images are reconstructed directly from PET raw data with well-modelled Poisson noise [15], whereas post-reconstruction dynamic PET data are used for the TAC-based analysis. We found, however, that the relative difference between multiparametric images and manually calculated MR_FDG_ was constant, and it is therefore our opinion that multiparametric MR_FDG_ values can be used with as much confidence as manually calculated values. The reproducibility of multiparametric images could be assessed in future test–retest studies.

Excluding the brain and the heart, surprisingly little data have been published on the TACs of FDG in normal tissues and tumours with the exception of some dual-time point studies [20–25]. With D-WB PET, TACs of tracer in all tissues within the entire body during the 60-min scan are available as opposed to the regular static PET scans. D-WB thus allows clinicians to perform a multi-time point type of analysis within the normal scan duration and for any area of the body. The TACs presented in this paper are in line with previous observations of mostly increasing FDG uptake in the heart and brain, and mostly decreasing uptake in the liver, kidneys, and the spleen. As such, these TACs are confirmatory, but they also attest to the ease with which TACs of pathologies ranging from inflammatory diseases to malignancies can be obtained.

A clear physiologic interpretation of parameters obtained from full kinetic modelling requires high-quality dynamic PET data and a physiologically reasonable model. Traditionally, kinetic modelling was limited to single organs that were analysed using dedicated organ- and tracer-specific models and input functions. D-WB FDG imaging has the great advantage of covering the larger axial field of view needed for multi-organ applications and metastatic disease evaluation in oncology, but in its current form the same model is applied to all tissues, which complicates reading of the multiparametric images. The Patlak model assumes an irreversible FDG uptake (trapped in the form of FDG-6-phosphate), and the model should be applied to D-WB data only after the blood pool and reversible compartments (free FDG in blood and tissue) reach a steady-state. These assumptions, which are clearly not met by all tissues, should be taken into account when interpreting multi-parametric images: liver and kidneys are examples of tissues that are better described by reversible kinetic models. However, it should be noted that WB parametric images provide MR_FDG_ and DV_FDG_ values for all tissues regardless of whether these tissues are characterized by irreversible FDG uptake. The resulting MR_FDG_ and DV_FDG_ values may therefore not always reflect an actual physiological estimate of metabolism.

Consequently, interpretation of multiparametric images requires insight into normal glucose metabolism in all organs and tissues. For example, glucose metabolism and consequently FDG uptake in the myocardium varies significantly between patients depending on, e.g. levels of insulin (high calorie versus low-calorie diets), length of fasting or previous exercise[26–28]. The large variability observed in cardiac MR_FDG_ values is therefore to be expected. Another particular case is the brain. For example, it is well known that SUV values in the brain decrease in patients with unregulated elevated blood sugar [18, 29]. This reduction in SUV (shown in Fig. 6A) occurs due to the competitive nature of glucose metabolism in the brain tissue and does not correspond to real metabolic changes in the brain, since brain glucose metabolism is not substrate driven. MR_FDG_ represents the actual metabolic rate of glucose in brain tissue, and the above correlations are therefore not present in the multiparametric images. Also of interest, and as demonstrated previously [30], the metabolic rate of glucose consumption in the brain was inversely correlated with age (Fig. 6C). Therefore, in patients with unregulated blood glucose levels, the use of multiparametric images could be advantageous over traditional SUV semi-quantification. Easy access to cross-tabulated MR_FDG_ in all tissues and organs also allows for hypothesis generating visualizations of organ cross-talk and the impact of, e.g. diabetes on glucose homeostasis (see Fig. 7).

Some limitations to the study and general use of D-WB should be noted. First, our study population consisted of patients referred for a FDG PET scan due to suspicion of pathology. Even though pathology was not always present, and no area of interest was drawn over areas visibly affected by disease, the measured normal values are not those of a healthy control population. However, our reported normal values were in line with results previously published in healthy volunteers for skeletal muscle [31, 32], the liver [33], the fasted heart [34, 35] and the brain [36, 37]. Second, current 70-min D-WB parametric protocols are laborious and occupy what is in all PET departments a finite amount of scanner time, which limits the everyday usability of this technique. However, by implementing population-based input functions, we expect that the length of the D-WB parametric imaging protocols can be reduced to 20 min, which allows a reasonable clinical throughput of two D-WB examinations per hour.

The study of whole-body metabolism is on path to make a resurgence in the nuclear medicine world. Total body PET scanners facilitate easier acquisition of whole body tracer kinetics and allow for more advanced modelling of tracer kinetics and organ interactions[38–41]. Upcoming parametric PET research could result in large libraries of human metabolic data, initially mostly based on FDG. Using FDG reference values and assisted by AI computing, clinicians may be able to interpret medical imaging in a way that was not possible before. For example, results of individual scans may be compared with values from a reference library of metabolic data in order to, e.g. modify individual treatment plans and dosages. However, this field of research should not be restricted to total body scanners, since even limited axial field of view PET scanners using automated whole-body parametric reconstruction algorithms can contribute valuable and reproducible data.

## Conclusion

The automated D-WB FDG scan protocol provides high-quality multiparametric images and organ MR_FDG_ values with little inter-subject variation and in agreement with manual TAC-based and literature values. The technique therefore facilitates multiparametric image reading, more accurate clinical reports and simpler acquisition of quantitative estimates of whole-body tissue glucose metabolism. Whole-body multi-parametric imaging can play an influential role in the future enhanced interpretation of PET images such as comparisons of individual patient data to multi-centre reference libraries of whole-body parametric images of biological relevant parameters and studies of interaction between organs.

## Supplementary Information

**Additional file 1. Table 1**. VOI delineation methodology. **Table 2A**. Normal values of males and females in the population without diabetes (N = 100). **Table 2B**. Normal values of males and females in the population with diabetes (N = 26). **Table 3**. Lumped Constant. **Figure 1**. Pearson’s correlation results for patients without diabetes. **Table 4A**. Patients without diabetes P values results for SUVmean. **Table 4B**. Patients without diabetes P values results for MRFDG mean. **Figure 2**. Pearson’s correlation results for patients with diabetes. **Table 5A**. Patients with diabetes P values results for SUVmean. **Table 5B**. Patients with diabetes P values results for MRFDG mean.

## Data Availability

Within the restrictions applied by the EU GDPR, all data are available from the authors upon reasonable request.

## Abbreviations

AI: Artificial intelligence
BMI: Body mass index
DM: Diabetes mellitus
DV_FDG_: Distribution volume of FDG
D-WB: Dynamic whole-body
FDG: ^18^F-fluorodeoxyglucose
FDG-6-P: FDG-6-phosphate
IDIF: Image-derived input function
MR_FDG_: Metabolic rate of FDG uptake
PET/CT: Positron emission tomography/computer tomography
SUV: Standard uptake value
TACs: Time-activity curves
