# Supplementary material for: Normal values for 18F-FDG uptake in organs and tissues measured by dynamic whole body multiparametric FDG PET in 126 patients
Source: EJNMMI Res. 2022 Mar 7;12:15. doi: 10.1186/s13550-022-00884-0 (PMC8901901; doi:10.1186/s13550-022-00884-0)
Supplement: Supplementary file 1 — Additional file 1. Table 1. VOI delineation methodology. Table 2A. Normal values of males and females in the population without diabetes (N = 100). Table 2B. Normal values of males and females in the population with diabetes (N = 26). Table 3. Lumped Constant. Figure 1. Pearson’s correlation results for patients without diabetes. Table 4A. Patients without diabetes P values results for SUVmean. Table 4B. Patients without diabetes P values results for MRFDG mean. Figure 2. Pearson’s correlation results for patients with diabetes. Table 5A. Patients with diabetes P values results for SUVmean. Table 5B. Patients with diabetes P values results for MRFDG mean. [file 13550_2022_884_MOESM1_ESM.pdf]

Normal values for ^{18}F-FDG uptake in organs and tissues measured by dynamic whole body multiparametric FDG PET in 126 patients

André H. Dias[1], MD, Allan K. Hansen, MD, PhD[1], Ole L. Munk[1,2], PhD, Lars C. Gormsen[1,2], MD, PhD

1 Department of Nuclear Medicine & PET Centre, Aarhus University Hospital, Denmark 2 Department of Clinical Medicine, Aarhus University; Denmark

Corresponding author: André H. Dias (andre.dias@auh.rm.dk)

SUPPLEMENTAL MATERIAL

Table 1: VOI delineation methodology

For all areas care was taken to ensure that the outlined VOIs corresponded to areas not affected by pathology	
**Brain (Grey and White matter)**	The [50-70min 6i/5s allpass reconstruction] was used for grey and white brain matter VOI definition. First, each subject's skull and brain were manually isolated using a box shaped VOI. Second, the isolated heads where rigidly matched to an FDG PET template included with PMOD. From these FDG PET heads in template space, tissue probability maps of white matter, grey matter and cerebrospinal fluid of each subject were generated using PMOD's adaption of the unified segmentation method from the SPM8 or SPM12 toolbox (https://www.fil.ion.ucl.ac.uk/spm/). The tool is named "MRI Probability / Inhomogeneity" in PMOD as it was developed for MRI images, however, it performs reasonably for FDG PET with carefully chosen cut-off values. The generated tissue probability maps were spatially transformed back into each subject's native space. Finally, individual VOIs were generated using a cutoff of >99.5% for gray matter probability and >90% for white matter. These values were chosen based on simple trial and error on the first few subjects. Ultimately, VOI definition in each subject was confirmed with visual inspection of VOIs overlayed on PET.
**Heart**	Manual delineation of a “horseshoe” shaped VOI over the area corresponding to the left ventricle of the heart. CT and dynamic imaging were used as reference to aid this procedure in patients with reduced left ventricle uptake.
**Bone**	Cubic VOI, 10 mm sides, placed centrally in the body of a healthy thoracal vertebra.
**Liver**	Spherical VOI, 30 mm in radius, placed centrally in an area of healthy hepatic tissue.
**Spleen**	Spherical VOI, 15 mm in radius, placed centrally in an area of healthy splenic tissue.

**Stomach**	Manually drawn VOI over the stomach area. PMOD's isocontour tool (40% Max) was then executed to obtain the final area of stomach wall to analyse.
**Lung**	Spherical VOI, 15 mm in radius, placed centrally in the right lung taking care to avoid overlap with bronchial structures.<br>It should be noted that with this VOI we are evaluating an organ with low tissue fraction due to the presence of air within the lung parenchyma. The resulting quantitative values should be interpreted accordingly.
**Pancreas**	Spherical VOI, 10 mm in radius, placed over the pancreas using both CT images and SUV signal as reference. PMOD's isocontour tool (40% Max) was then executed to obtain the final area of pancreatic tissue to analyse.
**Kidney**	Parenchymal areas in the upper pole of the kidney were manually delineated, avoiding areas corresponding to the kidney calyces. PMOD's isocontour tool (40% Max) was then executed to obtain the final area of kidney parenchyma to analyse.
**Colon**	Using CT images as a reference, we placed an oblong ovoid shape measuring approximately 37mm x 14mm x 19mm over a section of transverse colon. PMOD's isocontour tool (40% Max) was then executed to obtain the final area of colon tissue to analyse.
**Paravertebral Muscle**	Circular ROI, radius of 10mm, replicated downward in 20 consecutive slices in order to obtain a cylindrical VOI. This VOI was placed using CT images to avoid overlap with neighbouring bone structures.
**Thigh Muscle**	Circular ROI, radius of 15mm, replicated in 20 consecutive slices in order to obtain a cylindrical VOI. This VOI was placed using CT images to avoid overlap with neighbouring bone structures.

**Areas of Pathology**	Spherical VOI, adjusted in dimensions and contours to fit over the representative pathological area without overlapping neighbouring structures. PMOD's isocontour tool (40% Max) was then executed to obtain the final area to analyse.

TABLE 2A: Normal values of males and females in the population without diabetes (N=100)

Volume of Interest		SUV_{mean}* (g/mL)	SUV_{max}* (g/mL)	100 x MR_{FDG}* (µmol/g/min)	100 x K_i * (mL/mL/min)	DV_{FDG}* (%)
Bone	Male	1.89 [1.12-3.14]	2.86 [1.59-4.92]	3.81 [1.08-9.09]	0.68 [0.16-1.57]	32.58 [4.53-46.39]
	Female	1.91 [1.25-4.87]	2.60 [1.72-6.12]	3.66 [1.14-8.21]	0.63 [0.19-1.35]	29.67 [3.49-51]
Brain GM†	Male	7.73 [4.85-12.37]	15.19 [8.66-23.3]	17.33 [11.68-27.61]	3.31 [2.30-5.03]	74.56 [13.58-120.43]
	Female	8.47 [5.05-13.57]	15.23 [9.33-46.55]	17.91 [12.76-27.17]	3.18 [2.02-5.40]	85.74 [14.13-140.66]
Brain WM†	Male	3.36‡ [2.09-4.32]	5.79 [3.59-8.69]	5.98 [4.56-9.53]	1.09 [0.74-1.72]	46.37 [24.68-87.40]
	Female	3.57‡ [2.25-5.29]	5.98 [3.69-10.33]	6.42 [4.02-9.29]	1.08 [0.72-1.80]	45.97 [15.29-84.23]
Colon	Male	1.17 [0.52-6.36]	2.27 [0.95-12.25]	2.91 [1.54-21.23]	0.56 [0.23-3.60]	43.73 [24.75-174.41]
	Female	1.30 [0.79-3.70]	2.48 [1.56-6.94]	3.42 [1.24-9.32]	0.58 [0.24-1.58]	52.89 [17.45-109.59]
Heart	Male	4.28 [0.72-14.50]	9.93 [1.58-32.03]	11.25 [0.73-42.60]	1.59 [0.15-7.22]	49.64 [24.72-191.67]
	Female	4.80 [1.16-13.17]	9.46 [2.42-33.57]	11.03 [0.35-24.59]	1.86 [0.08-5.59]	49.75 [16.84-169.75]
Kidney	Male	2.02 [0.67-3.03]	3.56 [1.32-4.77]	3.97 [0.08-7.95]	0.70 [0.01-1.45]	100.18‡ [59.03-140.95]
	Female	2.03 [1.52-2.64]	3.52 [2.86-4.71]	3.66 [0.98-6.27]	0.67 [0.02-1.06]	86.96‡ [32.51-131.59]
Liver	Male	2.23 [1.31-2.87]	3.82 [2.58-5.19]	2.10 [0.74-4.26]	0.36 [0.12-0.73]	83.91 [63.86-126.02]
	Female	2.33 [1.71-3.11]	3.73 [2.51-5.69]	1.94 [0.82-4.35]	0.34 [0.16-0.70]	83.61 [43.66-103.93]
Lung	Male	0.40 [0.07-0.70]	0.86 [0.34-1.46]	0.37 [0.15-1.74]	0.68 [0.03-0.30]	14.53 [2.13-27.83]
	Female	0.42 [0.13-0.83]	0.82 [0.42-1.40]	0.32 [0.03-0.91]	0.06 [0.01-0.15]	15.57 [6.76-28.15]
Muscle Back	Male	0.57‡ [0.38-0.86]	1.02‡ [0.76-1.59]	0.81 [0.40-1.64]	0.14‡ [0.08-0.30]	13.83‡ [6.34-30.51]
	Female	0.71‡ [0.39-1.01]	1.19‡ [0.77-1.99]	0.95 [0.56-1.59]	0.17‡ [0.08-0.29]	18.13‡ [6.02-33.72]
Muscle Thigh	Male	0.50‡ [0.36-0.85]	1.13‡ [0.72-2.20]	0.84‡ [0.14-2.28]	0.15‡ [0.03-0.39]	12.73 [6.26-25.80]
	Female	0.62‡ [0.42-1.41]	1.28‡ [0.80-3.11]	1.03‡ [0.50-2.27]	0.18‡ [0.09-0.35]	13.72 [9.50-22.41]
Pancreas	Male	1.67 [1.15-2.44]	2.77 [1.64-4.29]	3.74‡ [2.01-6.67]	0.65‡ [0.34-1.15]	71.37‡ [42.68-123.80]
	Female	1.69 [1.18-2.38]	2.67 [2.01-3.81]	3.27‡ [1.16-5.77]	0.60‡ [0.26-0.94]	67.70‡ [42.57-84.65]
Spleen	Male	1.95 [1.41-4.95]	2.85 [2.08-6.37]	2.39 [1.31-15.30]	0.43 [0.24-2.78]	59.43‡ [40.23-83.99]
	Female	1.99 [1.50-5.93]	2.82 [1.98-8.46]	2.49 [1.18-9.65]	0.44 [0.20-1.72]	54.82‡ [10.07-111.59]
Stomach	Male	2.27 [1.33-4.08]	4.40 [2.43-7.99]	6.05‡ [2.92-19.13]	1.04‡ [0.49-3.24]	84.85‡ [48.53-178.42]
	Female	2.22 [1.43-5.25]	4.09 [2.55-9.72]	5.30‡ [2.84-12.18]	0.91‡ [0.64-2.03]	72.69‡ [45.22-168.43]

*****Values are median [min-max]; † Brain VOIs: N=61
‡ p-value <0.05 of T-Test male vs female
Note that MR_{FDG} and K_i values are multiplied by 100. Lumped constant=1.

TABLE 2B: Normal values of males and females in the population with diabetes (N=26)

Volume of Interest		SUV_{mean}* (g/mL)	SUV_{max}* (g/mL)	100 x MR_{FDG}* (μmol/g/min)	100 x K_i * (mL/mL/min)	DV_{FDG}* (%)
Bone	Male	1.83 [1.12-2.61]	2.72 [1.97-4.70]	1.50 [0.16-2.87]	0.68 [0.09-1.03]	25.04 [11.16-60.12]
	Female	2.01 [1.37-2.60]	2.92 [2.21-3.43]	1.51 [0.65-2.10]	0.49 [0.34-0.85]	21.79 [6.24-32.15]
Brain GM†	Male	5.07‡ [4.65-7.42]	2.79‡ [1.66-3.72]	4.24 [0.64-9.00]	2.04‡ [1.67-3.32]	66.45‡ [41.60-79.36]
	Female	8.17‡ [5.91-9.43]	2.72‡ [1.86-4.24]	4.35 [2.51-7.74]	2.41‡ [1.62-3.92]	72.68‡ [62.19-95.26]
Brain WM†	Male	2.50‡ [2.17-3.07]	9.79‡ [8.59-15.22]	16.00 [12.79-23.53]	0.84 [0.55-1.18]	39.67 [29.12-67.61]
	Female	3.41‡ [2.68-3.68]	14.83‡ [10.14-19.28]	19.14 [14.73-24.78]	0.89 [0.63-1.33]	39.86 [33.36-47.45]
Colon	Male	1.92 [0.87-5.72]	3.97 [3.63-5.49]	6.44 [4.75-7.35]	0.64 [0.10-1.61]	56.46 [35.80-99.15]
	Female	1.75 [1.27-2.63]	5.59 [4.39-6.68]	6.78 [4.20-8.26]	0.87 [0.44-2.19]	58.12 [39.37-163.69]
Heart	Male	2.51 [0.99-13.08]	3.25 [1.49-10.48]	6.07 [0.68-20.55]	0.80 [0.02-6.30]	47.43 [35.76-123.79]
	Female	3.34 [1.08-13.24]	3.08 [2.09-4.89]	6.05 [3.01-7.30]	0.86 [0.14-5.37]	51.62 [28.53-98.09]
Kidney	Male	2.29 [1.91-2.62]	6.99‡ [2.10-29.51]	5.65 [0.14-43.46]	0.68 [0.08-1.10]	105.12 [83.60-137.69]
	Female	2.41 [2.08-3.18]	7.35‡ [2.06-30.97]	6.31 [1.01-33.30]	0.67 [0.28-1.01]	90.74 [75.00-110.87]
Liver	Male	2.18 [1.77-2.62]	3.88 [3.11-4.64]	4.71 [0.53-8.38]	0.31 [0.03-0.54]	83.42 [62.71-105.89]
	Female	2.21 [1.86-2.81]	4.39 [3.56-6.05]	4.16 [2.20-6.25]	0.36 [0.31-0.51]	78.51 [69.32-89.32]
Lung	Male	0.38 [0.15-0.57]	3.84 [2.91-5.14]	2.76 [0.35-5.35]	0.06 [0.01-0.10]	15.56 [6.11-21.34]
	Female	0.43 [0.29-0.62]	3.86 [2.89-5.17]	2.54 [0.15-3.31]	0.07 [0.03-0.11]	14.30 [10.19-18.62]
Muscle Back	Male	0.63 [0.41-0.85]	0.75 [0.50-1.19]	0.51 [0.05-0.97]	0.17 [0.01-0.27]	15.89 [7.67-29.84]
	Female	0.70 [0.54-1.17]	0.87 [0.58-1.13]	0.52 [0.28-0.76]	0.13 [0.11-0.38]	16.38 [11.42-23.84]
Muscle Thigh	Male	0.51‡ [0.38-0.69]	1.18 [0.73-1.86]	1.24 [0.08-2.68]	0.14‡ [0.02-0.22]	15.95 [11.16-25.55]
	Female	0.64‡ [0.40-2.02]	1.41 [1.01-2.16]	1.15 [0.38-2.37]	0.14‡ [0.06-0.82]	18.83 [11.30-30.22]
Pancreas	Male	1.86 [1.50-2.41]	1.20 [0.81-1.57]	0.80 [0.10-1.66]	0.62 [0.12-0.80]	70.13 [42.48-92.01]
	Female	1.93 [1.29-2.43]	1.35 [0.92-4.39]	1.10 [0.47-5.11]	0.61 [0.45-0.73]	66.87 [50.20-88.54]
Spleen	Male	1.83 [1.55-2.16]	2.73 [2.07-3.43]	4.81 [0.81-6.06]	0.34 [0.05-0.62]	57.83 [41.57-87.58]
	Female	1.93 [1.54-2.20]	2.85 [2.04-3.17]	4.18 [0.89-6.09]	0.38 [0.24-0.56]	52.01 [43.18-67.48]
Stomach	Male	2.17 [1.47-2.82]	2.82 [2.27-3.69]	2.53 [0.37-5.41]	0.96 [0.14-1.17]	73.19 [58.65-97.16]
	Female	2.33 [1.98-2.72]	3.04 [2.19-3.23]	2.79 [1.07-3.68]	0.94 [0.68-1.41]	80.04 [40.73-102.84]

*****Values are median [min-max]; † Brain VOIs: N=14
‡ p-value <0.05 of T-Test male vs female
Note that MR_{FDG} and K_i values are multiplied by 100. Lumped constant=1.

Table 3: Lumped Constant

**The lumped constant of ^{18}F-FDG accounts for the differences in transport and phosphorylation rates between D-glucose and 2-fluoro-2-deoxy-D-glucose in different tissues, and is used to transform the ^{18}F-FDG uptake rate (MR_{FDG}) to glucose uptake rate.** $$MRglu = \frac{Cglu \times Ki}{LC}$$ **Although the LC is not known for most tissues, some publications have suggested the following values:**		
**Brain**	0.65 if irreversible uptake is assumed<br>0.81 if dephosphorylation is considered	Wu et al., 2003 (1)
	0.80	Graham et al., 2002 (2)
**Myocardium**	1.44 ±0.14 in the fasting state<br>0.99±0.07 during insulin infusion	Ng et al, 1998 (3)
**Muscle**	1.16	Peltoniemi et al., 2000 (4)
**Adipose tissue**	1.14	Virtanen et al., 2001 (5)
**Liver (in pigs)**	~1	Iozzo et al., 2007 (6)

Reference list

1. Wu H, Bergsneider M, Glenn T, Yeh E, Hovda D, Phelps M, et al. Measurement of the global lumped constant for 2-deoxy-2-[18F]fluoro-D-glucose in normal human brain using [15O]water and 2-deoxy-2-[18F]fluoro-D-glucose positron emission tomography imaging. A method with validation based on multiple methodologies. Molecular imaging and biology. 2003;5(1).
2. Graham M, Muzi M, Spence A, O'Sullivan F, Lewellen T, Link J, et al. The FDG lumped constant in normal human brain. Journal of nuclear medicine : official publication, Society of Nuclear Medicine. 2002;43(9).
3. Ng C, Soufer R, McNulty P. Effect of hyperinsulinemia on myocardial fluorine-18-FDG uptake. Journal of nuclear medicine : official publication, Society of Nuclear Medicine. 1998;39(3).
4. Peltoniemi P, Lönnroth P, Laine H, Oikonen V, Tolvanen T, Grönroos T, et al. Lumped constant for [(18)F]fluorodeoxyglucose in skeletal muscles of obese and nonobese humans. American journal of physiology Endocrinology and metabolism. 2000;279(5).
5. Virtanen K, Peltoniemi P, Marjamäki P, Asola M, Strindberg L, Parkkola R, et al. Human adipose tissue glucose uptake determined using [(18)F]-fluoro-deoxy-glucose ([(18)F]FDG) and PET in combination with microdialysis. Diabetologia. 2001;44(12).
6. Iozzo P, Jarvisalo M, Kiss J, Borra R, Naum G, Viljanen A, et al. Quantification of liver glucose metabolism by positron emission tomography: validation study in pigs. Gastroenterology. 2007;132(2).

Figure 1: Pearson's correlation results for patients without diabetes (DM). The value in each cell is the Pearson r value.

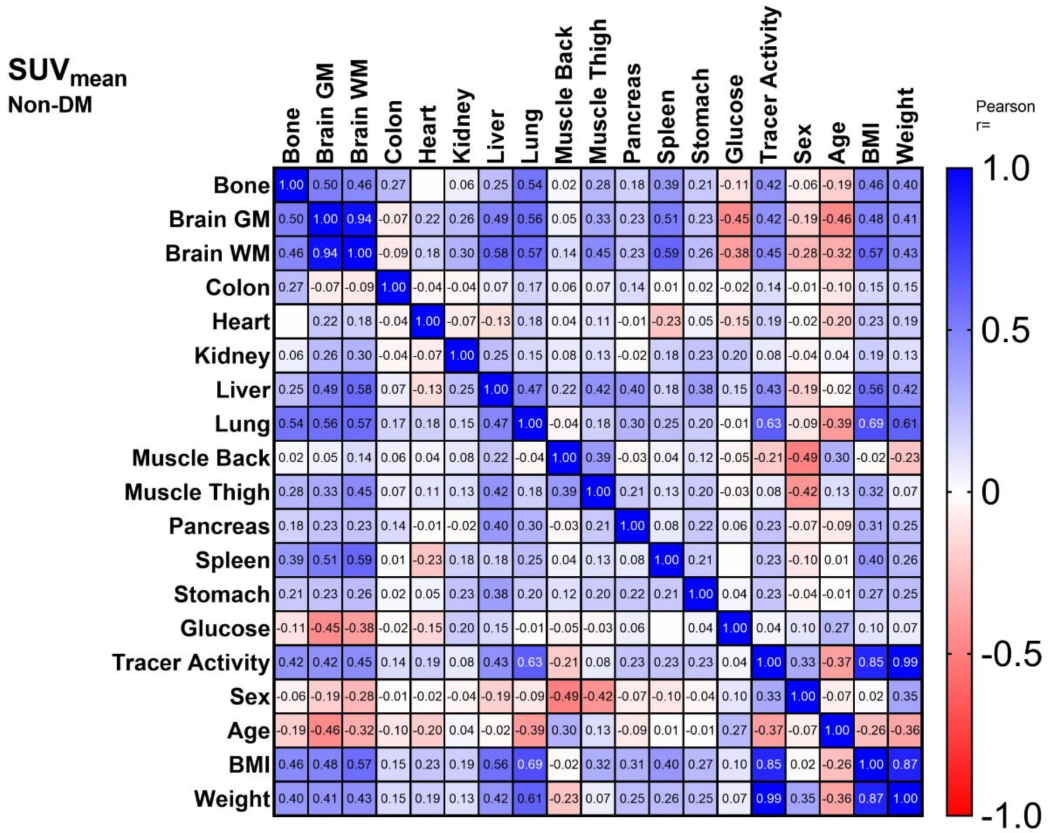

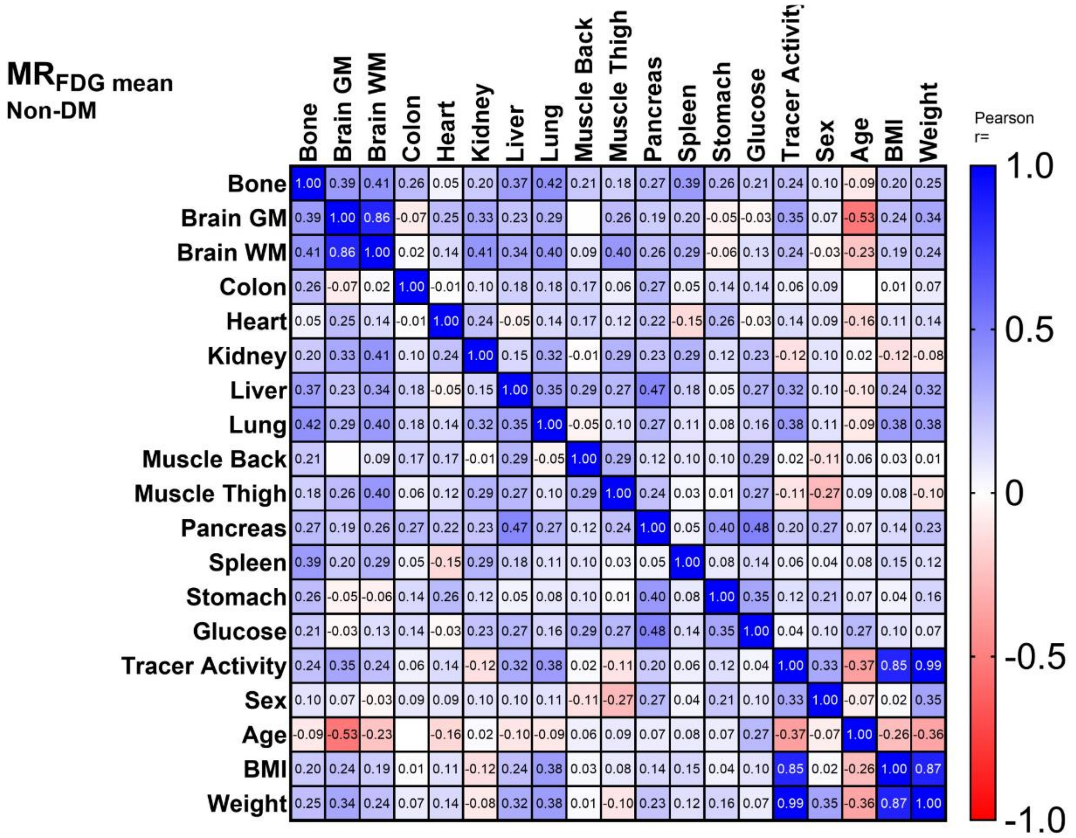

Table 4A: Patients without diabetes

SUVmean : P value results

SUV_{mean} Non-DM ***p values***	Bone	Brain GM	Brain WM	Colon	Heart	Kidney	Liver	Lung	Muscle Back	Muscle Thigh	Pancreas	Spleen	Stomach	Glucose	Tracer Activity	Sex	Age	BMI	Weight
Bone		<0.01	<0.01	0.01	0.99	0.55	0.01	<0.01	0.81	<0.01	0.07	<0.01	0.04	0.26	<0.01	0.56	0.06	<0.01	<0.01
Brain GM	<0.01		<0.01	0.57	0.10	0.04	<0.01	<0.01	0.69	0.01	0.07	<0.01	0.07	<0.01	<0.01	0.14	<0.01	<0.01	<0.01
Brain WM	<0.01	<0.01		0.50	0.17	0.02	<0.01	<0.01	0.29	<0.01	0.07	<0.01	0.04	<0.01	<0.01	0.03	0.01	<0.01	<0.01
Colon	0.01	0.57	0.50		0.69	0.72	0.50	0.09	0.55	0.50	0.17	0.93	0.81	0.85	0.18	0.89	0.34	0.13	0.14
Heart	0.99	0.10	0.17	0.69		0.48	0.20	0.07	0.71	0.29	0.89	0.02	0.65	0.14	0.05	0.86	0.05	0.02	0.05
Kidney	0.55	0.04	0.02	0.72	0.48		0.01	0.13	0.41	0.20	0.82	0.08	0.02	0.04	0.43	0.67	0.71	0.06	0.21
Liver	0.01	<0.01	<0.01	0.50	0.20	0.01		<0.01	0.03	<0.01	<0.01	0.08	<0.01	0.15	<0.01	0.06	0.81	<0.01	<0.01
Lung	<0.01	<0.01	<0.01	0.09	0.07	0.13	0.00		0.73	0.07	<0.01	0.01	0.05	0.92	<0.01	0.36	<0.01	<0.01	<0.01
Muscle Back	0.81	0.69	0.29	0.55	0.71	0.41	0.03	0.73		<0.01	0.77	0.68	0.25	0.65	0.04	<0.01	<0.01	0.84	0.02
Muscle Thigh	<0.01	0.01	<0.01	0.50	0.29	0.20	<0.01	0.07	<0.01		0.04	0.21	0.05	0.73	0.42	<0.01	0.19	<0.01	0.51
Pancreas	0.07	0.07	0.07	0.17	0.89	0.82	<0.01	<0.01	0.77	0.04		0.40	0.03	0.53	0.02	0.47	0.37	<0.01	0.01
Spleen	<0.01	<0.01	<0.01	0.93	0.02	0.08	0.08	0.01	0.68	0.21	0.40		0.04	0.98	0.02	0.33	0.92	<0.01	0.01
Stomach	0.04	0.07	0.04	0.81	0.65	0.02	<0.01	0.05	0.25	0.05	0.03	0.04		0.71	0.02	0.70	0.93	0.01	0.01
Glucose	0.26	<0.01	<0.01	0.85	0.14	0.04	0.15	0.92	0.65	0.73	0.53	0.98	0.71		0.71	0.32	0.01	0.31	0.47
Tracer Activity	<0.01	<0.01	<0.01	0.18	0.05	0.43	<0.01	<0.01	0.04	0.42	0.02	0.02	0.02	0.71		0.00	<0.01	<0.01	<0.01
Sex	0.56	0.14	0.03	0.89	0.86	0.67	0.06	0.36	<0.01	<0.01	0.47	0.33	0.70	0.32	<0.01		0.50	0.86	<0.01
Age	0.06	<0.01	0.01	0.34	0.05	0.71	0.81	<0.01	<0.01	0.19	0.37	0.92	0.93	0.01	<0.01	0.50		0.01	<0.01
BMI	<0.01	<0.01	<0.01	0.13	0.02	0.06	<0.01	<0.01	0.84	<0.01	<0.01	<0.01	0.01	0.31	<0.01	0.86	0.01		<0.01
Weight	<0.01	<0.01	<0.01	0.14	0.05	0.21	<0.01	<0.01	0.02	0.51	0.01	0.01	0.01	0.47	<0.01	<0.01	<0.01	0.00	

Table 4B: Patients without diabetes

MR$_{FDG\ mean}$: P value results

MR$_{FDG}$ Non-DM *p values*	Bone	Brain GM	Brain WM	Colon	Heart	Kidney	Liver	Lung	Muscle Back	Muscle Thigh	Pancreas	Spleen	Stomach	Glucose	Tracer Activity	Sex	Age	BMI	Weight
Bone		<0.01	<0.01	0.01	0.64	0.04	<0.01	<0.01	0.04	0.07	0.01	<0.01	0.01	0.04	0.02	0.31	0.39	0.04	0.01
Brain GM	<0.01		<0.01	0.57	0.05	0.01	0.08	0.02	0.99	0.04	0.13	0.13	0.71	0.82	0.01	0.59	<0.01	0.06	0.01
Brain WM	<0.01	<0.01		0.89	0.28	0.00	0.01	<0.01	0.47	<0.01	0.04	0.02	0.67	0.33	0.06	0.79	0.07	0.14	0.06
Colon	0.01	0.57	0.89		0.95	0.33	0.08	0.08	0.09	0.53	0.01	0.63	0.15	0.17	0.58	0.39	1.00	0.91	0.48
Heart	0.64	0.05	0.28	0.95		0.02	0.64	0.15	0.09	0.25	0.03	0.15	0.01	0.76	0.18	0.36	0.12	0.27	0.16
Kidney	0.04	0.01	<0.01	0.33	0.02		0.14	<0.01	0.91	<0.01	0.02	<0.01	0.22	0.02	0.24	0.33	0.86	0.22	0.41
Liver	<0.01	0.08	0.01	0.08	0.64	0.14		<0.01	<0.01	0.01	<0.01	0.08	0.65	0.01	<0.01	0.31	0.34	0.02	<0.01
Lung	<0.01	0.02	<0.01	0.08	0.15	<0.01	<0.01		0.66	0.30	0.01	0.28	0.43	0.11	<0.01	0.29	0.37	<0.01	<0.01
Muscle Back	0.04	0.99	0.47	0.09	0.09	0.91	<0.01	0.66		<0.01	0.23	0.32	0.34	<0.01	0.88	0.26	0.58	0.79	0.88
Muscle Thigh	0.07	0.04	0.00	0.53	0.25	<0.01	0.01	0.30	0.00		0.02	0.76	0.90	0.01	0.28	0.01	0.40	0.41	0.33
Pancreas	0.01	0.13	0.04	0.01	0.03	0.02	<0.01	0.01	0.23	0.02		0.65	0.00	0.00	0.04	0.01	0.50	0.17	0.02
Spleen	<0.01	0.13	0.02	0.63	0.15	<0.01	0.08	0.28	0.32	0.76	0.65		0.42	0.18	0.55	0.71	0.44	0.15	0.25
Stomach	0.01	0.71	0.67	0.15	0.01	0.22	0.65	0.43	0.34	0.90	<0.01	0.42		<0.01	0.23	0.04	0.49	0.67	0.11
Glucose	0.04	0.82	0.33	0.17	0.76	0.02	0.01	0.11	<0.01	0.01	<0.01	0.18	<0.01		0.71	0.32	0.01	0.31	0.47
Tracer Activity	0.02	0.01	0.06	0.58	0.18	0.24	<0.01	<0.01	0.88	0.28	0.04	0.55	0.23	0.71		<0.01	<0.01	<0.01	<0.01
Sex	0.31	0.59	0.79	0.39	0.36	0.33	0.31	0.29	0.26	0.01	0.01	0.71	0.04	0.32	<0.01		0.50	0.86	<0.01
Age	0.39	<0.01	0.07	1.00	0.12	0.86	0.34	0.37	0.58	0.40	0.50	0.44	0.49	0.01	<0.01	0.50		0.01	<0.01
BMI	0.04	0.06	0.14	0.91	0.27	0.22	0.02	<0.01	0.79	0.41	0.17	0.15	0.67	0.31	<0.01	0.86	0.01		<0.01
Weight	0.01	0.01	0.06	0.48	0.16	0.41	<0.01	<0.01	0.88	0.33	0.02	0.25	0.11	0.47	<0.01	<0.01	<0.01	<0.01	

Figure 2: Pearson's correlation results for patients with diabetes. The value in each cell is the Pearson r value.

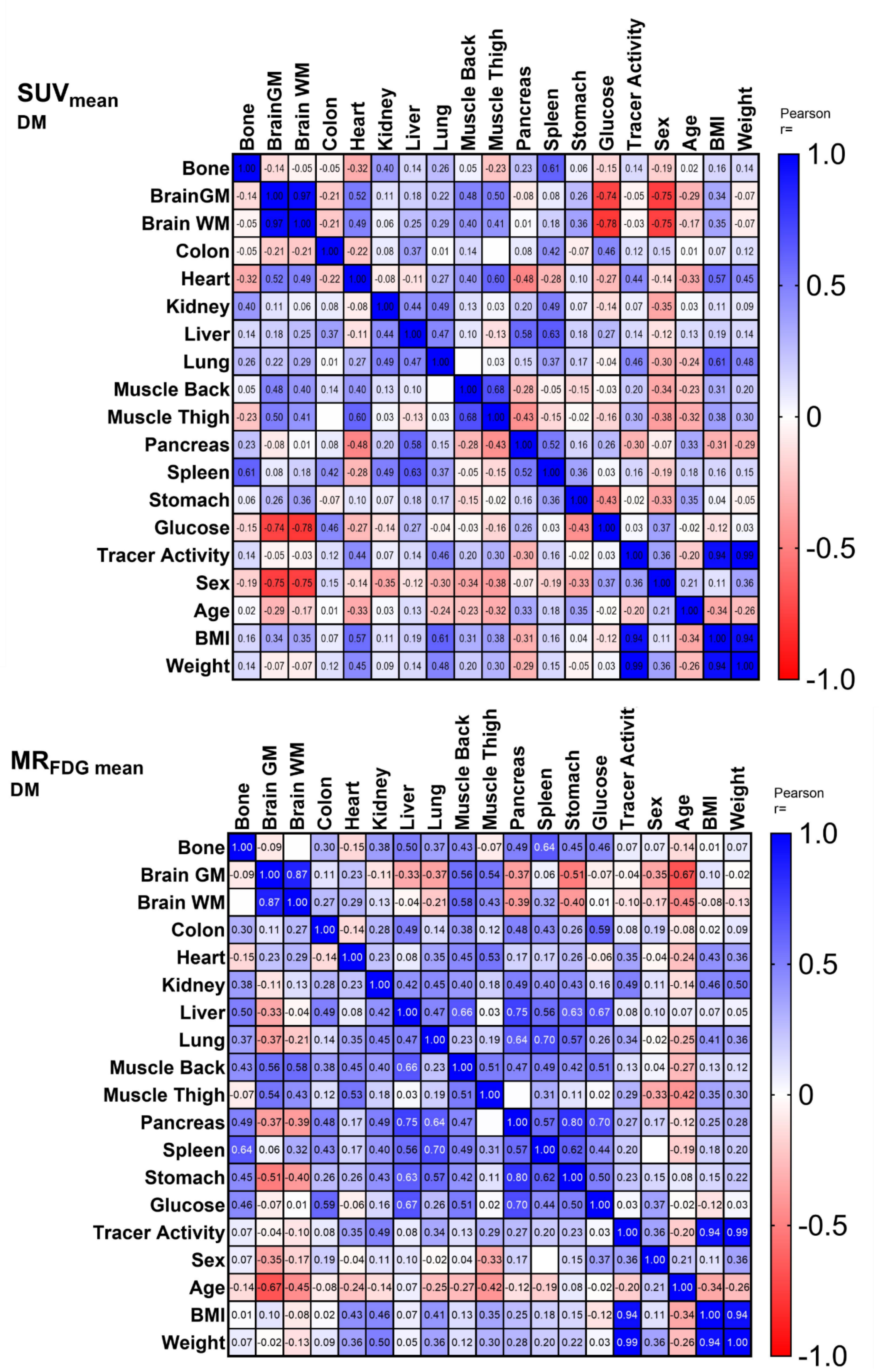

Table 5A: Patients with diabetes
SUV_{mean}: P value results

**SUVmean** **DM** ***p values***	Bone	Brain GM	Brain WM	Colon	Heart	Kidney	Liver	Lung	Muscle Back	Muscle Thigh	Pancreas	Spleen	Stomach	Glucose	Tracer Activity	Sex	Age	BMI	Weight
Bone		0.63	0.88	0.83	0.11	0.04	0.49	0.20	0.80	0.25	0.25	<0.01	0.76	0.46	0.49	0.36	0.92	0.44	0.50
Brain GM	0.63		<0.01	0.48	0.06	0.70	0.53	0.45	0.08	0.07	0.77	0.80	0.37	<0.01	0.87	<0.01	0.31	0.23	0.81
Brain WM	0.88	<0.01		0.47	0.08	0.85	0.39	0.31	0.15	0.14	0.96	0.54	0.21	<0.01	0.91	<0.01	0.56	0.22	0.80
Colon	0.83	0.48	0.47		0.29	0.70	0.06	0.96	0.50	1.00	0.72	0.03	0.73	0.02	0.54	0.47	0.97	0.72	0.56
Heart	0.11	0.06	0.08	0.29		0.71	0.59	0.19	0.04	<0.01	0.01	0.17	0.63	0.18	0.02	0.50	0.10	0.00	0.02
Kidney	0.04	0.70	0.85	0.70	0.71		0.03	0.01	0.54	0.87	0.33	0.01	0.73	0.51	0.75	0.08	0.90	0.58	0.67
Liver	0.49	0.53	0.39	0.06	0.59	0.03		0.02	0.63	0.54	<0.01	<0.01	0.38	0.19	0.49	0.57	0.53	0.35	0.48
Lung	0.20	0.45	0.31	0.96	0.19	0.01	0.02		0.98	0.90	0.46	0.07	0.40	0.84	0.02	0.14	0.24	<0.01	0.01
Muscle Back	0.80	0.08	0.15	0.50	0.04	0.54	0.63	0.98		0.00	0.17	0.79	0.46	0.88	0.33	0.09	0.25	0.12	0.33
Muscle Thigh	0.25	0.07	0.14	1.00	<0.01	0.87	0.54	0.90	0.00		0.03	0.47	0.92	0.43	0.14	0.05	0.11	0.06	0.14
Pancreas	0.25	0.77	0.96	0.72	0.01	0.33	<0.01	0.46	0.17	0.03		0.01	0.44	0.19	0.14	0.74	0.10	0.12	0.14
Spleen	<0.01	0.80	0.54	0.03	0.17	0.01	<0.01	0.07	0.79	0.47	0.01		0.07	0.90	0.43	0.36	0.39	0.44	0.48
Stomach	0.76	0.37	0.21	0.73	0.63	0.73	0.38	0.40	0.46	0.92	0.44	0.07		0.03	0.94	0.09	0.08	0.85	0.82
Glucose	0.46	0.00	0.00	0.02	0.18	0.51	0.19	0.84	0.88	0.43	0.19	0.90	0.03		0.89	0.06	0.94	0.57	0.87
Tracer Activity	0.49	0.87	0.91	0.54	0.02	0.75	0.49	0.02	0.33	0.14	0.14	0.43	0.94	0.89		0.07	0.33	0.00	<0.01
Sex	0.36	<0.01	<0.01	0.47	0.50	0.08	0.57	0.14	0.09	0.05	0.74	0.36	0.09	0.06	0.07		0.31	0.58	0.07
Age	0.92	0.31	0.56	0.97	0.10	0.90	0.53	0.24	0.25	0.11	0.10	0.39	0.08	0.94	0.33	0.31		0.09	0.19
BMI	0.44	0.23	0.22	0.72	<0.01	0.58	0.35	<0.01	0.12	0.06	0.12	0.44	0.85	0.57	0.00	0.58	0.09		0.00
Weight	0.50	0.81	0.80	0.56	0.02	0.67	0.48	0.01	0.33	0.14	0.14	0.48	0.82	0.87	0.00	0.07	0.19	<0.01	

Table 5B: Patients with diabetes
MR$_{FDG\ mean}$: P value results

**MR$_{FDG}$ DM *p values***	Bone	Brain GM	Brain WM	Colon	Heart	Kidney	Liver	Lung	Muscle Back	Muscle Thigh	Pancreas	Spleen	Stomach	Glucose	Tracer Activity	Sex	Age	BMI	Weight
Bone		0.77	0.99	0.14	0.46	0.06	0.01	0.06	0.03	0.73	0.01	<0.01	0.02	0.02	0.73	0.73	0.50	0.96	0.72
Brain GM	0.77		<0.01	0.71	0.42	0.71	0.24	0.20	0.04	0.05	0.19	0.84	0.06	0.81	0.89	0.22	0.01	0.72	0.96
Brain WM	0.99	<0.01		0.35	0.32	0.67	0.90	0.46	0.03	0.12	0.17	0.27	0.16	0.98	0.72	0.56	0.11	0.78	0.66
Colon	0.14	0.71	0.35		0.49	0.17	0.01	0.50	0.05	0.56	0.01	0.03	0.20	<0.01	0.69	0.36	0.69	0.93	0.65
Heart	0.46	0.42	0.32	0.49		0.25	0.71	0.08	0.02	0.00	0.40	0.40	0.20	0.76	0.08	0.85	0.23	0.03	0.07
Kidney	0.06	0.71	0.67	0.17	0.25		0.03	0.02	0.05	0.37	0.01	0.04	0.03	0.45	0.01	0.59	0.51	0.02	0.01
Liver	0.01	0.24	0.90	0.01	0.71	0.03		0.01	<0.01	0.90	<0.01	<0.01	<0.01	<0.01	0.70	0.62	0.75	0.74	0.80
Lung	0.06	0.20	0.46	0.50	0.08	0.02	0.01		0.25	0.35	<0.01	<0.01	<0.01	0.20	0.09	0.93	0.21	0.04	0.07
Muscle Back	0.03	0.04	0.03	0.05	0.02	0.05	0.00	0.25		0.01	0.02	0.01	0.03	0.01	0.53	0.85	0.19	0.52	0.55
Muscle Thigh	0.73	0.05	0.12	0.56	<0.01	0.37	0.90	0.35	0.01		0.98	0.12	0.59	0.91	0.14	0.10	0.03	0.08	0.14
Pancreas	0.01	0.19	0.17	0.01	0.40	0.01	<0.01	<0.01	0.02	0.98		0.00	<0.01	<0.01	0.18	0.40	0.55	0.21	0.16
Spleen	<0.01	0.84	0.27	0.03	0.40	0.04	<0.01	<0.01	0.01	0.12	<0.01		<0.01	0.02	0.33	0.99	0.34	0.38	0.33
Stomach	0.02	0.06	0.16	0.20	0.20	0.03	<0.01	<0.01	0.03	0.59	<0.01	<0.01		0.01	0.27	0.45	0.69	0.45	0.28
Glucose	0.02	0.81	0.98	<0.01	0.76	0.45	<0.01	0.20	0.01	0.91	<0.01	0.02	0.01		0.89	0.06	0.94	0.57	0.87
Tracer Activity	0.73	0.89	0.72	0.69	0.08	0.01	0.70	0.09	0.53	0.14	0.18	0.33	0.27	0.89		0.07	0.33	0.00	<0.01
Sex	0.73	0.22	0.56	0.36	0.85	0.59	0.62	0.93	0.85	0.10	0.40	0.99	0.45	0.06	0.07		0.31	0.58	0.07
Age	0.50	0.01	0.11	0.69	0.23	0.51	0.75	0.21	0.19	0.03	0.55	0.34	0.69	0.94	0.33	0.31		0.09	0.19
BMI	0.96	0.72	0.78	0.93	0.03	0.02	0.74	0.04	0.52	0.08	0.21	0.38	0.45	0.57	0.00	0.58	0.09		<0.01
Weight	0.72	0.96	0.66	0.65	0.07	0.01	0.80	0.07	0.55	0.14	0.16	0.33	0.28	0.87	0.00	0.07	0.19	<0.01	
